# A National Dietary Assessment Reference Database (NDARD) for the Dutch Population: Rationale behind the Design

**DOI:** 10.3390/nu9101136

**Published:** 2017-10-18

**Authors:** Elske M. Brouwer-Brolsma, Martinette T. Streppel, Linde van Lee, Anouk Geelen, Diewertje Sluik, Anne M. van de Wiel, Jeanne H. M. de Vries, Pieter van ’t Veer, Edith J. M. Feskens

**Affiliations:** Division of Human Nutrition, Wageningen University, 6700 AA Wageningen, The Netherlands; elske.brouwer-brolsma@wur.nl (E.M.B.-B.); m.t.streppel@hva.nl (M.T.S.); Linde_Van_Lee@sics.a-star.edu.sg (L.v.L.); Anouk.geelen@wur.nl (A.G.); Diewertje.sluik@wur.nl (D.S.); anne.vandewiel@wur.nl (A.M.v.d.W.); Jeanne.devries@wur.nl (J.H.M.d.V.); Pieter.vantveer@wur.nl (P.v.V.)

**Keywords:** dietary assessment, FFQ, recall, nutritional biomarker, validation

## Abstract

The development of reliable Food Frequency Questionnaires (FFQs) requires detailed information about the level and variation of dietary intake of the target population. However, these data are often limited. To facilitate the development of new high quality FFQs and validation of existing FFQs, we developed a comprehensive National Dietary Assessment Reference Database (NDARD) detailing information about the level and variation in dietary intake of people 20–70 years old in the general Dutch population. This paper describes the methods and characteristics of the population included in the NDARD database. A total of 1063 men and 985 women agreed to participate in this study. Dietary intake data were collected using different FFQs, web-based and telephone-based 24-h recalls, as well as blood and urine-based biomarkers. The baseline FFQ was completed by 1647 participants with a mean age of 51 ± 12 years, BMI of 26 ± 4 kg/m^2^, and energy intake of 2051 ± 605 kcal/day. The percentage of total energy intake from proteins, carbohydrates, and fats were 15 ± 2, 43 ± 6, and 36 ± 5 En%, respectively. A total of 1113 participants completed telephone-based recalls and 1783 participants completed web-based recalls. This database will enable researchers to validate existing national FFQs and to develop new high quality dietary assessment methods.

## 1. Introduction

To study the impact of diet on disease risk in observational studies, it is crucial to obtain valid information about the habitual dietary intake of the population under study. Since food frequency questionnaires (FFQs) are relatively easy and inexpensive to process [1], large-scale epidemiological studies generally use FFQs to rank participants according to their nutrient or food intake [1]. However, it is challenging to develop a valid and reliable FFQ.

The first challenge in developing an FFQ is to accurately identify food items that contain the nutrients to be studied in the target population. In the Netherlands, researchers currently use the results of the Dutch National Food Consumption Survey (DNFCS), compiled from a representative sample of the Dutch population [2], to identify relevant food items [3]. One of the drawbacks of using the DNFCS data is that they are collected by means of duplicate 24-h recalls [2] and thus provide limited information on the day-to-day variation in dietary intake. Therefore, the DNFCS data are considered to be insufficient to develop FFQs since it does not represent the actual large within-person day-to-day variation of nutrients such as vitamin A, vitamin C, and cholesterol [1].

A second challenge arises when assessing the validity and reproducibility of the FFQ. FFQs are often validated using other dietary assessment methods, such as 24-h recalls, as the reference method. One major drawback of this approach is that the results of the validation study may be biased by correlated errors such as memory, use of the same food composition tables, and/or use of standard portion sizes [4]. A biomarker of food intake is considered to be a more independent reference method for the validation of an FFQ since this method has fewer correlated errors. Unfortunately, food biomarkers are only available for a limited number of nutrients/foods. Urinary nitrogen, potassium, sodium, and doubly labelled water are examples of validated recovery markers/techniques that are used to estimate absolute intakes [5]. Carotenoids and *n*-3 fatty acids are validated markers to rank people according to their fruit/vegetable and fish intake [6,7]. 

Ideally, FFQs should be validated for each nutrient under investigation. However, since validation studies are cumbersome, costly, and inefficient, many of the nutrients studied by FFQs are not validated. In the absence of a validation study, misclassification of nutrient intake may remain unnoticed and result in flawed conclusions with respect to potential diet–disease relationships. Thus, it is clear that the validation of a FFQ is very important and that the simplification of the validation process would greatly benefit studies investigating the effect of diet on disease risk. 

We believe that a database containing extensive FFQ data, data from multiple 24-h recalls as well as biomarkers, would provide the unique opportunity to perform validation studies on existing FFQs that are cheaper and less time consuming than the validation studies that are currently conducted in the context of individual studies. Moreover, such a database would also benefit the development and validation of new FFQs. Therefore, we initiated the National Dietary Assessment Reference Database (NDARD) project and developed a national database providing data on nutrient levels and variations in habitual dietary intake as assessed by FFQs, multiple 24-h recalls, and biomarkers. This database can (1) serve as the foundation for the development of new dietary assessment methods; and (2) facilitate the validation of existing and newly developed FFQs. With this manuscript, we aim to describe the methods used to collect the NDARD data and its population characteristics.

## 2. Materials and Methods 

### 2.1. Design 

The NDARD database contains data from 2048 men and women aged 20 to 70 years collected between May 2011 and February 2013, living in and around the city of Wageningen in the Netherlands. Municipality registries from Ede, Wageningen, Renkum, and Arnhem were used to select eligible participants. In addition, all households in the city of Veenendaal received an invitation to participate as well. To be eligible, participants had to be able to speak and write Dutch. Those who were interested were asked to register online. Once registered, eligible participants were invited to the study center and randomly assigned to either the ‘FFQ group’ (*n* = 959) or the ‘24-h recall group’ (*n* = 1089). All participants—i.e., participants in the ‘FFQ group’ and participants in the ‘24-h recall group’—completed a general 183-item FFQ. Thereafter, data collection in the ‘FFQ group’ focused on the validation of an unconventional FFQ: the Flower FFQ. In the ‘24-h recall group’, dietary data collection primarily focused on the collection of repeated telephone-based and web-based 24-h recalls. Along with the dietary intake assessment, participants underwent anthropometric measurements and a venipuncture, collected 24-h urine samples, and completed questionnaires related to their health and lifestyle. Measurements were repeated after 12 and 24 months (Table 1). All participants provided written informed consent before commencing the study. The study was approved by the ethical committee of Wageningen University and conducted according to the declaration of Helsinki.

### 2.2. Dietary Intake Assessment

#### 2.2.1. General FFQ

All participants completed a 183-item semiquantitative general FFQ, which was designed shortly before the start of the study. This FFQ was designed to cover ≥96% of the absolute level of food intake and ≥95% of the between-person variability of each nutrient under study as assessed in the DNFCS from 1998 [8]. Commonly eaten manufactured food products that appeared on the market later than 1998 were selected from the Dutch National Food Consumption Survey of 2011 and included in the FFQ as well. The reference period of the FFQ was the previous month. Participants answered questions relating to frequency by selecting answers ranging from ‘never’ to ‘6–7 days per week’. Portion sizes were estimated using natural portions and commonly used household measures. Average daily nutrient intakes were calculated by multiplying the consumption frequency by the portion size and nutrient content in grams as indicated in the most recent Dutch food composition table (2011) [9]. Intake levels for energy, macronutrients, dietary fiber, and selected vitamins were validated [10,11,12]. The FFQ was self-administered and completed online (open-source survey tool LimeSurvey™, LimeSurvey Project Team/Carsten Schmitz, Hamburg, Germany). Trained research dieticians conducted several quality checks to ensure the quality of the FFQ.

#### 2.2.2. Flower FFQ

Participants assigned to the FFQ group also completed a new type of FFQ, the Flower FFQ. The Flower FFQ was developed for the LifeLines Cohort Study [13] as an alternative to the regular FFQ, which is often a long and time-consuming questionnaire. The name Flower FFQ has been derived from its design. The questionnaire consists of one basic questionnaire about energy and macronutrient intake (the heart of the flower) and three complementary food questionnaires concerning specific (micro)nutrients (flower petals). The basic FFQ contains 110 food items that are used to estimate the intakes of energy, fat, carbohydrates, protein, and alcohol. The first complementary FFQ (Flower special FFQ1) contains 59 food items and is used to estimate the intake levels of different types of fatty acids as well as caffeine. The Flower special FFQ2 consists of 61 food items and is used to estimate the intake levels of vitamin B_2_, vitamin B_6_, vitamin B_11_, vitamin B_12_, calcium, and soy. Lastly, the Flower special FFQ3 consists of 64 food items and is used to estimate the intake levels of vitamin A, vitamin C, vitamin E, and dietary fiber. Thus, the difference between the FFQs is in the degree of detail requested. For instance, the basic FFQ provides information on the amount of bread consumed, but does not contain questions about the type of bread consumed. More detailed information about types of bread consumed—including fiber intake—has to be obtained from the flower special FFQ3, where the question “How many slices of bread do you eat?” is followed by questions on the type of bread: white, whole grain, etc. To match the basic FFQ with the flower specials, the flower specials also contain the general questions that overlap with the basic questionnaire. In order to calculate the food and nutrient intakes, information in the basic FFQ was assumed to be superior. More specifically, if a food was reported in the basic FFQ, but not in the flower special, a weighted average for the particular food subgroups was assigned. If a food was not reported in the basic FFQ while it was reported in flower special, the food was recorded as not consumed. Combined, the four FFQs cover ≥96% of the absolute nutrient intake and ≥93% of the between-person variability of each nutrient as assessed in the DNFCS from 1998. Participants answered questions on frequency by selecting answers ranging from ‘never’ to ‘6–7 days per week’. Portion sizes were estimated using natural portions and commonly used household measures. Average daily nutrient intakes were calculated by multiplying consumption frequency by portion size and nutrient content per gram as indicated in the Dutch food composition table from 2006 [14]. Although the reference period of the FFQs is one month, it is assumed that food consumption patterns are stable over a longer period of time. Therefore, the four flower FFQs can be completed at different moments during a study period. In view of the validation studies within the NDARD-project all flower FFQs were administered three times (i.e., repeated after six and 12 months), which offers the opportunity to study reproducibility as well as seasonal influences. The Flower FFQ was administered online via the open-source survey tool LimeSurvey™, Hamburg, Germany.

#### 2.2.3. Telephone-Based and Web-Based 24-h Recalls

Participants in the ‘recall-group’ were invited to complete nine 24-h recalls during a period of one year, where the minimum period between the completion of two recalls was at least two weeks. Three of nine 24-h recalls were telephone-based and six recalls were web-based. The dates for either telephone-based or web-based 24-h recalls were randomly selected, scheduled regularly throughout the year, and proportionally evenly distributed over weekdays (±70%) and weekend days (±30%). The mode of administration, i.e., via telephone or internet, was also randomly selected. When recall attempts or electronic invitations were denied, the recall was randomly rescheduled within three to 10 days. Telephone-based 24-h recalls were performed by trained dieticians using a standardized protocol and conducted using the five-step multiple pass method, which is a validated technique that increases the accuracy of recalls [15,16,17,18]. Portion sizes were assessed using commonly used household measures, weight/volume, and standard portions. The recalls were transcribed into the food codes of the Dutch food composition table of 2011 [9]. 

All dieticians participated in regular meetings to conduct quality checks to ensure the quality of phone-based recalls and data encoding. Web-based recalls were self-administered using the software program Compl-eat (www.compleat.nl). Compl-eat was developed based on the five-step multiple pass method [15,16,17,18], which guided the participant through the process of reporting all foods and drinks consumed during the previous day. The program enabled participants to select standard foods and recipes that are commonly used in the Netherlands. If necessary, participants were able to adapt or describe personal recipes or to make notes for clarification. Portion sizes were reported in commonly used household measures, standard portions, and weight in grams or volume in liters. Invitations to complete a 24-h recall were sent randomly via e-mail and valid for 24 h upon receipt. When participants did not respond within 24 h, the computer randomly generated a new invitation within 3–10 days after the first invitation. This process continued until the participant completed the recall. Nutrient and energy intakes were again calculated by multiplying intakes by nutrient composition using the Dutch food composition database from 2011 [9].

Furthermore, the intake of dietary supplements or the need to follow a particular diet, prescribed or otherwise, were registered. Participants also indicated where they ate their principal meals (location) and if they were eating alone or accompanied by others (companionship). Additional information such as the need to follow a particular diet or the occurrence of special occasions such as birthdays or holidays were taken into consideration while checking the recalls. Participants were not contacted for clarifications in case of potential errors in the dietary data. However, trained dieticians did check all 24-h recalls for completeness, unusual portion sizes, and notes. Errors and notes were corrected according to a standardized approach using standard portion sizes and recipes. For instance, if a participant reported to consume 125 cups of coffee, this was corrected to 1 cup of 125 g. 

### 2.3. Anthropometric Measurements

Anthropometric examinations were conducted by well-trained staff according to a standardized protocol. Height was measured, without shoes, with a stadiometer (SECA, Hamburg, Germany) to the nearest 0.1 cm. Weight was measured, without shoes or sweaters and with empty pockets, on a digital scale (SECA, Hamburg, Germany) to the nearest 0.1 kg. Waist and hip circumferences were measured twice to the nearest 0.5 cm using a measuring tape (SECA 201, Hamburg, Germany) and subsequently averaged. Waist circumference was measured midway between the lowest rib and the top of the iliac crest at the end of gentle expiration. Hip circumference was measured around the widest portion of the buttocks. During the anthropometric examination, information about medication and nutritional supplement use was collected as well. The type of medication was classified according to the Anatomical Therapeutic Chemical classification system.

### 2.4. Blood Collection

After a 10-h overnight fast participants underwent a venipuncture at the Gelderse Vallei hospital in Ede or at the Rijnstate hospital in Velp. Biochemical analyses were performed in the hospital laboratories using either a Dimension Vista 1500 automated analyzer (Siemens, Erlangen, Germany) or a Roche Modular P800 chemistry analyzer (Roche Diagnostics, Indianapolis, IN, USA). Both laboratories join the external quality control program in the Netherlands (SKML) and use the same methodology and standardized protocols for risk factor assessments. Blood samples were used to determine carotenoid and *n*-3 fatty acid concentrations, which can be used as a reference for ranking based on fruit and vegetable intake [6] and fish intake [7]. The remaining plasma and serum samples have been stored at −80 °C until further analysis is needed.

### 2.5. Urine Collection

Participants were asked to collect urine during one 24-h period each year. The day prior to the planned 24-h urine collection participants received verbal and written instructions, three 80 mg para-aminobenzoic acid (PABA) tablets (PABAcheck, Elsie Widdowson Laboratory, Cambridge, UK), and two three-liter containers containing the preservative lithium dihydrogenphosphate (25 g). Urine collection started after the first voiding after waking up and finished after the first voiding after waking up 24-h later. Participants were instructed to record the beginning and end times of the urine collection, the time at which the PABA tablets were ingested, their medication and nutritional supplement use, and any possible deviations from the protocol (e.g., missing urine). Urine containers were delivered to the Gelderse Vallei hospital in Ede or the Rijnstate hospital in Velp and were stored at 4 °C for a maximum of three days until they could be transported to the study center. At the study center, the urine collections of each participant were mixed, weighted, aliquoted, and stored at −20 °C until further analyses. PABA was used to check the completeness of the urinary collections and was measured using the HPLC method [19]. PABA is assumed to be excreted almost quantitatively within 24-h. Therefore, a recovery of at least 78% (189.6 mg) of the ingested PABA was considered to be a complete urine collection. The total coefficient of variation (CV) for the PABA analysis was 9%. The within-run CV for PABA was 1.9% and the between-run CV for PABA was 1.3%. Urinary sodium and potassium concentrations were measured with an ion-selective electrode on a Roche 917 analyzer (Roche Diagnostics, Indianapolis, IN, USA). Urinary creatinine concentrations were measured at 520 nm on the Synchron LX20 by the modified Jaffé procedure using a commercial kit. Total 24-h sodium and potassium excretions were calculated by multiplying the total weight of the collected urine by the sodium or potassium concentration. Additionally, this was divided by 0.86 for sodium [20] and by 0.81 for potassium [21], assuming that this percentage of intake is excreted in the urine. The total 24-h nitrogen excretion was determined by the Foss Kjeltec^TM^ 2300 analyzer [22]. The urinary nitrogen level was subsequently calculated using the following formula: 6.25x (urinary nitrogen/0.81) [23], which takes into account nitrogen loss via feces and skin (approximately 19%). Three 24-h 4.5 mL urine samples have been stored at −20 °C in the NDARD biobank for future analyses.

### 2.6. Health and Lifestyle Questionnaires

Health and lifestyle questionnaires were completed online using LimeSurvey™, Hamburg, Germany. The questionnaires included questions about demographics (e.g., birth country, marital status, household composition, and education), work history and current work situation, health and history of diseases, and current and previous smoking habits (e.g., amount smoked, age at the beginning and the end of smoking periods and the type of tobacco smoked as well as passive smoking). Specifically for education, participants with no, primary, or lower vocational education were categorized as low educated. Participants with lower secondary or intermediate vocational education were categorized as intermediate educated. Participants with higher secondary education, higher vocational education, or university were categorized as high educated. Regarding smoking, the category “never smokers” exists of participants who did not smoke during the past month and also never smoked for a full year. Thus, this category may include participants that smoke rarely, e.g., at a party. “Current smokers” smoked during the past month or at some point smoked for a full year and did not stop smoking. “Former smokers” exists of those who at some point smoked for a full year, but did not smoke during the past month and stopped smoking. These general questions were predominantly derived from LifeLines study questionnaires [13]. 

Information about the habitual physical activity level was assessed using the Short Questionnaire to Assess Health-enhancing Physical Activity (SQUASH) and the Activity Questionnaire for Adults and Adolescents (AQuAA). The SQUASH contains separate questions about commuting activities, leisure time activities, household activities, and activities at work and school covering three main queries: days per week, average time per day, and intensity. The total minutes of activity were calculated for each question by multiplying frequency by duration. The activity scores for separate questions were calculated by multiplying total minutes of activity by the intensity score. The total activity score was calculated by taking the sum of the activity scores for separate questions [24]. The AQuAA is based on the SQUASH questionnaire with the following adaptations: questions on light, moderate, and vigorous intensity activities as well as sedentary behaviors; questions about age-specific examples of activities; questions relating to activities performed in the previous seven days instead of an average week. The main outcomes are total physical activity score and the time spent on sedentary, light, moderate and vigorous intensity activities in minutes per week [25]. All questionnaires were repeated after 12 and 24 months. 

## 3. Results

Table 2 displays the numbers and proportions of participants providing anthropometric data, blood samples, and urine samples. Moreover, the numbers and proportions of participants completing demographic/lifestyle, health and dietary questionnaires are presented. Anthropometric data were available of 2047 (100%) participants. In total 1881 (92%) participants provided blood and urine samples. Health and demographic/lifestyle questionnaires were completed by 1955 (95%) and 2038 (100%) participants, respectively. General FFQ data were available of 1647 (80%) participants. In the FFQ group, 53 (0%) participants completed 1.1 ± 0.2 (mean ± SD) telephone-based 24-h recalls and 832 (87%) participants completed 4.0±1.7 web-based 24-h recalls. Moreover, 772 (81%) participants completed the basic FFQ of the flower FFQ, 709 (74%) completed the flower special FFQ1, 593 (62%) completed the flower special FFQ2, and 558 (58%) completed the flower special FFQ3. In the recall group, 1060 (97%) participants completed 3.8 ± 1.5 telephone-based 24-h recalls and 951 (87%) participants completed 6.2 ± 2.6 web-based 24-h recalls.

In Table 3, general characteristics of the NDARD population are shown. Men and women were fairly equally represented, 52% vs. 48%. On average, participants were 51 ± 12 years of age, 63% were classified as having a high educational level, and 9% claimed to be current smokers. The mean BMI was 26 ± 4 kg/m^2^, where the BMI for men was slightly higher than that for women. 

Table 4 displays the habitual dietary intake of the NDARD population and DNFCS population. The average energy intake can be broken down into total protein intake 15 ± 2%, total carbohydrate intake 43 ± 6%, total fat intake was 36 ± 5%, and total dietary fiber intake 24 ± 7 g per day. There were no large differences in macronutrient intakes between men and women. However, men consumed more alcohol, 15 ± 15 g, than women, 7 ± 9 g. Nine percent of the participants reported to follow a special diet (7% of the men and 12% of the women), and 41% reported nutritional supplement use (34% men, 49% women).

## 4. Discussion

The NDARD project is a four-year longitudinal study that collected an extensive body of data on habitual dietary intake among 2048 Dutch adults living in and around the city of Wageningen in the Netherlands. These data are meant to serve as the basis for the development of a national dietary reference database that can be used for the development of novel FFQs and the validation of existing and newly developed FFQs. 

A major asset of the NDARD database is it contains data obtained by FFQs, multiple 24-h recalls, and biochemical markers—all covering a similar time window. This allows the validation of FFQs against multiple recalls, recovery markers, and concentration markers [26]. The first validation studies using the NDARD database have been published [27,28,29] and ongoing work addresses the validation of the Flower FFQ. In addition, a national FFQ for the Netherlands, the FFQ-NL1.0, has been validated in a subsample of 450 participants examining multiple 24-h recalls, urinary nitrogen and potassium, and plasma concentrations of fatty acids and carotenoids [30]. These examples illustrate that the NDARD is already a well-functioning reference database to conduct validation studies. Apart from concurrent validation, we can use NDARD for ‘backward’ validation of existing FFQs and ‘forward’ validation of FFQ-like instruments under construction for prioritized nutrients and/or food habits. Dutch FFQs are generally developed from 24-h recalls with food codes detailed to the level of the Dutch food composition table (NEVO-table). To come to FFQ-items, these food codes are grouped together and their nutrient composition is obtained as a weighted average of the intake of the underlying items based on 24-h recalls [31] available from, e.g., national food consumption surveys [2]. Subsequently, the nutrient intake according to the FFQ is simulated based on various options for grouping into FFQ-items and compared to the original 24-h recall intake and biomarker data in the NDARD. Thus, depending on their objectives, FFQs differ in their grouping of food codes into FFQ-items; grouping highly different composed foods into FFQ-items increases the systematic and random between-person errors in estimated nutrient intake, known to be relatively large in FFQs. As errors in memory and response may also be related to combining foods into items, such simulations could help to identify which potential FFQs would not perform well due to unfavorably composed items. If this approach appears rather robust, unnecessary costs due to data collection, biochemical analyses, and reference methods (24-h recalls, dairies) can be avoided or reduced in future studies.

In this paper, we focus on the methods of the ‘basic’ sample of the NDARD project that comprises data from 2048 Dutch adults, aged 20–70 years, living in the central part of the Netherlands. The nutritional data described in the results section of this paper includes data that were collected using a general FFQ and completed by both the recall group as well as the FFQ group. As shown in the results section, the dietary intake of the NDARD population closely resembles the dietary intake of the DNFCS population from 2007 to 2010 [2]. For instance, according to FFQ data, the breakdown of the total energy intake for women in the NDARD population was 15 ± 2% of energy from protein, 43 ± 6% of energy from carbohydrates, and 36 ± 6% of energy from fat. Correspondingly, in the DNFCS study from 2007 to 2010, the food intakes of these macronutrients for women aged 19–69 years were around 16% from protein, 45% from carbohydrates, and 34% from fat. The data on alcohol intake was similar when comparing the data collected in the NDARD database as compared to the DNFCS database. Fiber intake was somewhat higher in the NDARD population (mean 24 g) than in the DNFCS (median 18–23 g). As in the NDARD population, no major differences were observed in macronutrient intake between men and women in the DNFCS. Alcohol intake seemed to be only exception to this. 

As the DNFCS aims to recruit a study population representative of the Dutch adult population, comparison of the NDARD characteristics with the DNFCS characteristics gives an impression of the generalizability of our data. On the one hand, when looking at the proportion of men and women and the BMI, we can conclude that these characteristics are rather comparable between the two populations. On the other hand, participants in the NDARD project are relatively highly educated and less likely to be smoking compared to participants in the DNFCS. However, we do not expect that these factors will influence the validation studies that will be conducted using the NDARD database. In fact, due to their higher educational level, NDARD participants may have been even more thorough and precise in completing the study questionnaires compared to the general population. Nevertheless, it needs to be emphasized that the NDARD database cannot be used for the validation or development of FFQs for children and adolescents. Moreover, non-Dutch speaking migrants and illiterate persons were not included in the study population. In order to generate an even more comprehensive database, we plan to include partner studies. 

In conclusion, in the absence of a gold standard reference database, the evaluation and validation of FFQs is currently very time-consuming and expensive. The NDARD database now provides researchers with the opportunity to conduct more accurate and cost and time effective validation studies of existing FFQs and to develop new, high quality dietary assessment methods.

## Figures and Tables

**Table 1:** Overview of measurements of the National Dietary Assessment Reference Database (NDARD) project.

	Months						
Measurement	0	6	12	18	24	30	36
*All subjects (n = 2048)*							
Anthropometric measurements	x		x		x		
Blood collection	x		x		x		
24-h urine collection	x		x		x		
Health questionnaires	x		x		x		
Demographic/lifestyle questionnaires	x		x		x		
*FFQ group (n = 959)*							
General FFQ	x						
Flower basic FFQ	x	x	x				
Flower special FFQ1			x	x	x		
Flower special FFQ2					x	x	x
Flower special FFQ3					x	x	x
24-h recall (web based)	x	x	x	x	x	x	
*Recall group (n = 1089)*							
General FFQ	x		x		x		
24-h phone based recall	x	x	x		x		x
24-h web based recall	x	x	x		x		x

FFQ: food frequency questionnaire.

**Table 2:** Sample sizes (*n*) of the data collected in view of the NDARD project.

	All (*n* = 2048)	Men (*n* = 1063)	Women (*n* = 985)	FFQ Group (*n* = 959)	Recall Group (*n* = 1089)
Complete data collection, *n* (%)					
Anthropometric measures	2047 (100)	1062 (100)	985 (100)	959 (100)	1088 (100)
Blood samples	1881 (92)	959 (90)	922 (94)	886 (92)	995 (91)
Urine samples	1881 (92)	959 (90)	922 (94)	883 (92)	998 (92)
Health questionnaires	1955 (95)	1005 (95)	950 (96)	920 (96)	1035 (95)
Demographic and lifestyle questionnaires	2038 (100)	1060 (100)	978 (99)	954 (99)	1084 (100)
General FFQ	1647 (80)	857 (81)	790 (80)	666 (69)	981 (90)
Flower basic FFQ	772 (38)	404 (38)	368 (37)	772 (81)	-
Flower special FFQ1	709 (35)	353 (33)	356 (36)	709 (74)	-
Flower special FFQ2	593 (29)	296 (28)	297 (30)	593 (62)	-
Flower special FFQ3	558 (27)	277 (26)	281 (29)	558 (58)	-
Web based 24-h recall	1783 (87)	920 (87)	863 (88)	832 (87)	951 (87)
Phone-based 24-h recall	1113 (54)	585 (55)	528 (54)	53 (0)	1060 (97)

**Table 3:** Comparison of the participant characteristics of the NDARD project (*n* = 2048) and the Dutch National Food Consumption Survey (DNFCS) (*n* = 2106).

	*n*	All	Men	Women	FFQ Group	Recall Group	DNFCS 19–69 Year *
Men, %	2048	52	100	0	50	53	50
Age (years), mean (SD) **	2045	51 (12)	54 (12)	49 (13)	51 (13)	52 (12)	-
Education level, %	2038						
Low		7	9	6	7	7	32
Intermediate		30	28	32	31	30	45
High		63	63	62	62	63	23
Area, %	2048						
Ede/Wageningen/Renkum		45	32	60	52	40	-
Arnhem		11	7	15	11	11	-
Veenendaal		44	61	25	37	49	-
Smoking status, %	1541						
Current		9	10	8	9	10	25
Former		40	45	34	40	39	32
Never		51	45	58	51	51	43
BMI (kg/m^2^), mean (SD)	2047	26 (4)	27 (4)	26 (5)	26 (4)	26 (4)	26 (-)
Waist (cm), mean (SD)	2044	92 (13)	97 (11)	86 (12)	92 (13)	92 (13)	-
Disease history, %							
Myocardial infarction	1945	2	3	1	2	2	-
Stroke	1946	1	1	1	1	1	-
Diabetes mellitus	1955	4	5	2	4	4	-
Cancer	1949	5	5	6	5	5	-
ALT (U/L), mean (SD)	1879	27.0 (14.9)	31.3 (15.2)	22.5 (13.2)	27.5 (16.4)	26.5 (13.1)	-
AST (U/L), mean (SD)	1878	22.8 (8.5)	23.6 (8.7)	21.9 (8.3)	22.6 (9.2)	23.0 (7.6)	-
GGT (U/L), mean (SD)	1881	24.8 (25.2)	29.7 (28.8)	19.8 (19.6)	24.8 (26.1)	25.0 (24.2)	-
GFR (mL/min/1.73 m^2^), mean (SD)	1881	89.9 (14.6)	88.4 (14.3)	91.2 (14.8)	89.3 (14.1)	90.3 (15.2)	-

* The DNFCS does not present data for the total group. Therefore, values are calculated using available subgroup data. Moreover, not all data is available in the DNFCS report, which is indicated by “-”. ** In the DNFCS only age ranges per subgroup are provided. ALT: Alanine aminotransferase; AST: Aspartate aminotransferase; GGT: Gamma-glutamyltransferase; GFR: Glomerular filtration rate.

**Table 4:** Comparison of the dietary intake of the NDARD participants, as assessed with a general Food Frequency Questionnaires (FFQ), and the DNFCS.

Dietary Factor	NDARD All	NDARD Men	NDARD Women	NDARD FFQ Group	NDARD Recall Group	DNFCS Men 31–50 Year *	DNFCS Women 31–50 Year *
N	1647	857	790	666	981	348	351
Total energy (kcal), mean (SD)	2051 (605)	2244 (631)	1842 (499)	2033 (623)	2064 (593)	2647 (2299–3022)	1956 (1700–2227)
Total protein (energy%), mean (SD)	15 (2)	15 (2)	15 (2)	15 (2)	15 (2)	15 (14–17)	16 (14–17)
Total carbohydrates (energy%), mean (SD)	43 (6)	43 (6)	43 (6)	43 (6)	43 (6)	43 (40–47)	45 (42–49)
Mono- and disaccharides (energy%), mean (SD)	19 (5)	18 (5)	20 (5)	19 (5)	19 (5)	-	-
Polysaccharides (% of energy), mean (SD)	24 (5)	25 (4)	24 (5)	24 (5)	24 (5)	-	-
Total fat, energy%	36 (5)	36 (5)	36 (6)	36 (5)	36 (6)	35 (32–37)	34 (31–37)
Saturated fatty acids (energy%), mean (SD)	12 (3)	12 (3)	12 (3)	12 (3)	12 (3)	13 (11–14)	13 (11–14)
Monounsaturated fatty acids (energy%), mean (SD)	13 (2)	13 (2)	13 (3)	13 (2)	13 (2)	-	-
Polyunsaturated fatty acids (energy%), mean (SD)	8 (2)	8 (2)	8 (2)	8 (2)	8 (2)	7 (6–8)	7 (6–8)
Trans fatty acids (energy%), mean (SD)	0.6 (0.2)	0.6 (0.2)	0.5 (0.2)	0.5 (0.2)	0.6 (0.2)	1.5 (1.2–2.0)	1.2 (0.9–1.6)
Alcohol (gram), mean (SD)	11 (13)	15 (15)	7 (9)	12 (14)	11 (12)	16 (6–29)	4 (1–11)
Dietary fiber (gram), mean (SD)	24 (7)	25 (8)	23 (7)	24 (8)	24 (7)	23 (19–27)	18 (15–22)
Following diet regimen during the past month, %	9	7	12	10	9	9	19
Nutritional supplement use, %	41	34	49	38	43	36	54

* The DNFCS does not display means but medians (25th–75th percentiles). In addition, in the DNFCS, data is presented by subgroups for age. In this table, intakes for men and women aged 31–50 years are displayed.
